# Supplementary material for: Aerosol delivery of SARS-CoV-2 human monoclonal antibodies in macaques limits viral replication and lung pathology
Source: Nat Commun. 2023 Nov 3;14:7062. doi: 10.1038/s41467-023-42440-x (PMC10624670; doi:10.1038/s41467-023-42440-x)
Supplement: Supplementary file 1 — Supplementary Information [file 41467_2023_42440_MOESM1_ESM.pdf]

Supplementary Data

Aerosol Delivery of SARS-CoV-2 Human Monoclonal Antibodies in Macaques Limits Viral Replication and Lung Pathology

Daniel N. Streblow Ph.D.[a], Alec J. Hirsch Ph.D.[a], Jeffrey J. Stanton D.V.M.[b], Anne D. Lewis D.V.M., Ph.D., DACVP [b], Lois Colgin D.V.M. , DACVP [b], Ann J. Hessell Ph.D.[b], Craig N. Kreklywich B.S. [a], Jessica L. Smith Ph.D.[a], William F. Sutton M.S.[b], David Chauvin, Ph.D. [c], Jennifer Woo, D.V.M, Ph.D. [c], Benjamin N. Bimber Ph.D.[b], Cierra N. LeBlanc B.S. [d], Sonia N. Acharya Ph.D. [d], Brian J. O'Roak Ph.D.[d], Harjinder Sardar Ph.D.[e], Mohammad M. Sajadi M.D.[f], Zahra R. Tehrani Ph.D.[g], Mark R. Walter, Ph.D.[h], Luis Martinez-Sobrido, Ph.D.[i], James J. Kobie Ph.D.[j], Rachel J. Reader Ph.D. DACVP [k], Katherine J. Olstad D.V.M. DACVP [k], Theodore R. Hobbs D.V.M.[b], Erica Ollmann Saphire Ph.D.[l], Sharon L. Schendel Ph.D. [l], Robert H. Carnahan Ph.D.[m], Jonas Knoch M.Sc.[n], Luis M. Branco, Ph.D.[o], James E. Crowe, Jr. M.D. [m], Koen K.A. Van Rompay D.V.M., Ph.D. [k], Phillip Lovalenti Ph.D.[c], Vu Truong Ph.D.[c*], Donald N. Forthal M.D. [p*], Nancy L. Haigwood Ph.D. [b*]

SUPPLEMENTARY FIGURES AND TABLES

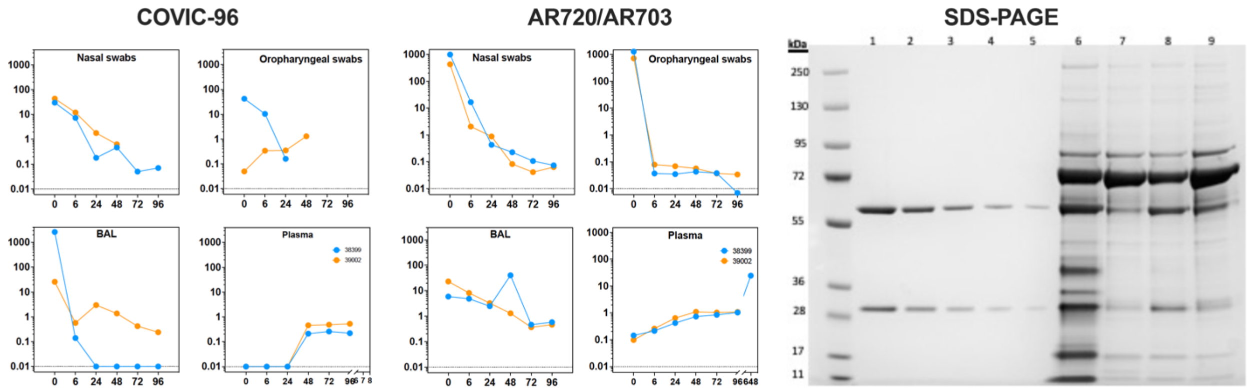

Supplementary Figure 1. Administration of inhaled, nebulized mAb in uninfected macaques. Qualitative measures of mAb concentrations as a function of time in fluids of macaques receiving nebulized mAbs. Concentrations of CoVIC-96 (left 4 panels) and ART720/AR703 (center 4 panels) in BAL, NAS, OAS, and blood plasma were quantified from clarified samples from each animal in the group (n=2) collected 0, 6, 24, 48, 72 and 96 hours post-treatment using antigen-specific quantitative ELISA. The colors blue and orange indicate samples from the two different animals in each group. ELISA assays were repeated at least twice with closely similar results. The right hand panel shows an SDS-PAGE gel stained with Novex SimplyBlue colloidal stain, showing anti-SARS-CoV-2 mAb combination standard loaded onto the gel (lanes 1-5 at 2, 1, 0.5, 0.25, and 0.125 µg) and BAL samples from macaques given the mAb combination at a dose of 37.5 mg/kg. Animal 38399, 0 h after aerosol delivery (lane 6) and 6 h after delivery (lane 7). Animal 39002, 0 h after aerosol delivery (lane 8) and 6 h after delivery (lane 9). Stained gels were repeated twice with similar results.

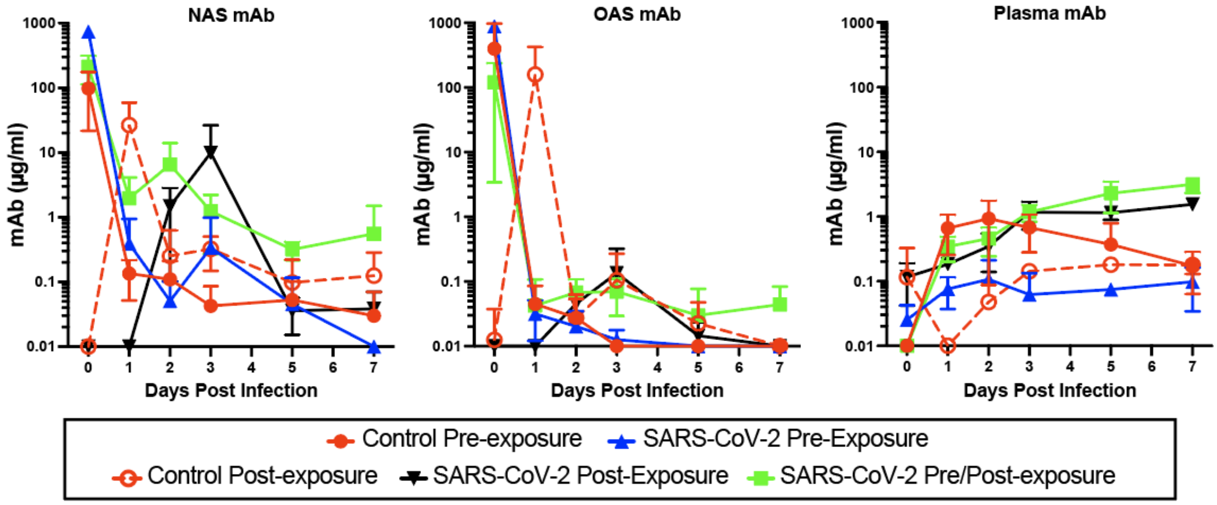

Supplementary Figure 2. Detection of inhaled, nebulized mAbs in NAS, OAS and blood plasma. Qualitative measures of control anti-RSV and anti-SARS-CoV-2 ART720/AR703 mAbs in NAS, OAS, and blood plasma were determined from clarified samples from each animal in the group (n=4 animals/group) collected at 0, 1, 3, 5, and 7 dpi using antigen specific quantitative ELISA. Graphs are labeled at the top by sample type tested. Individual data points for each animal within each group are shown as the mean and SEM for each group. The key for colors and symbols is provided below the graphs.

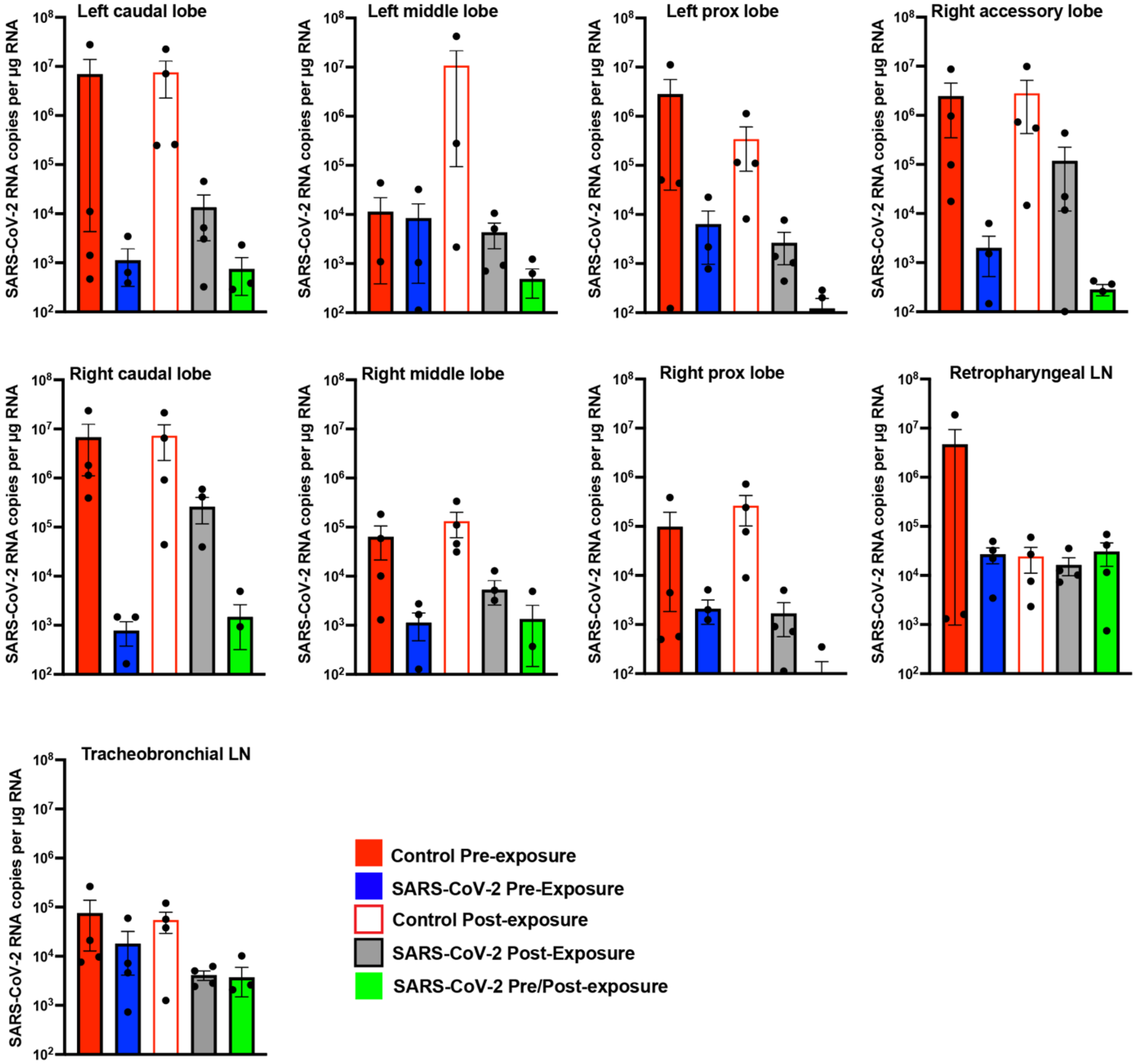

Supplementary Figure 3. Inhaled aerosolized Anti-SARS-CoV-2 mAb treatment blocks SARS-CoV-2 replication in respiratory tissues. RNA was extracted from respiratory tract tissues and viral RNA levels were determined by qRT-PCR using SARS-CoV-2-specific primers and probes. Individual data points are shown for each animal within each group (n=4 animals per group) and the mean and SEM for each group is indicated. The identity of each bar is indicated by a key, shown next to the graph.

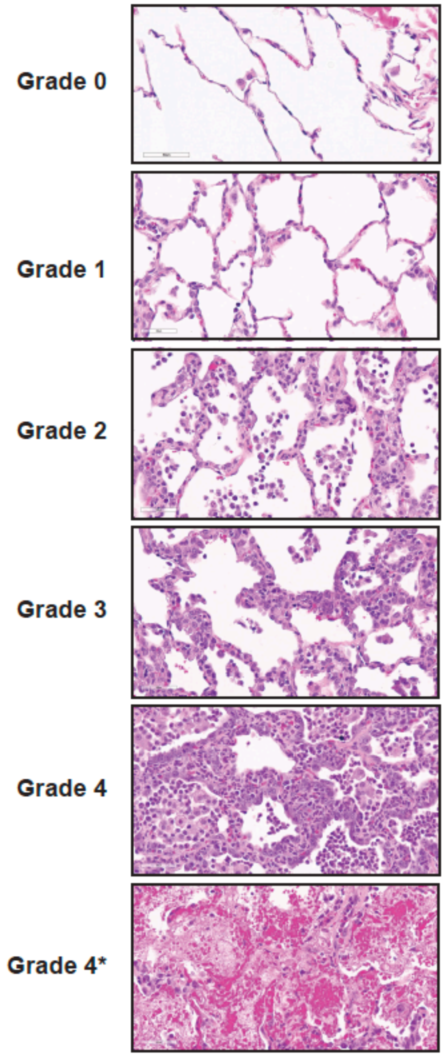

Supplementary Figure 4. Lung pathology scoring system. Slides containing section(s) of lung were scanned at 40x with an Aperio AT2 Leica Biosystems microscope slide scanner. A semiquantitative, scoring system was developed by assessing the interstitial cellularity of the alveolar septa. An initial score reflecting the most severely affected area was assigned to each lung lobe evaluated and illustrated in the photomicrographs. The scoring system was as follows: 0 = Normal cellularity; 1 = 1-2 cells thick; 2 = 2-4 cells thick; 3 = 4-6 cells thick; and 4 = > 6 cells thick or necrohemorrhagic lesions (Van Rompay et al. 2022[1]). The percentage of the tissue exhibiting any degree of increased cellularity and inflammation was estimated. Final scores for each lung lobe were obtained by multiplying the initial scores by the percentage of the tissue sections affected. If the percentage of the lung affected was less than 10%, a final score of 0 was assigned. An average score for each animal was calculated by combining the final scores of all lung lobes divided by the number of slides evaluated. Scale bar = 50 µm for all pictures.

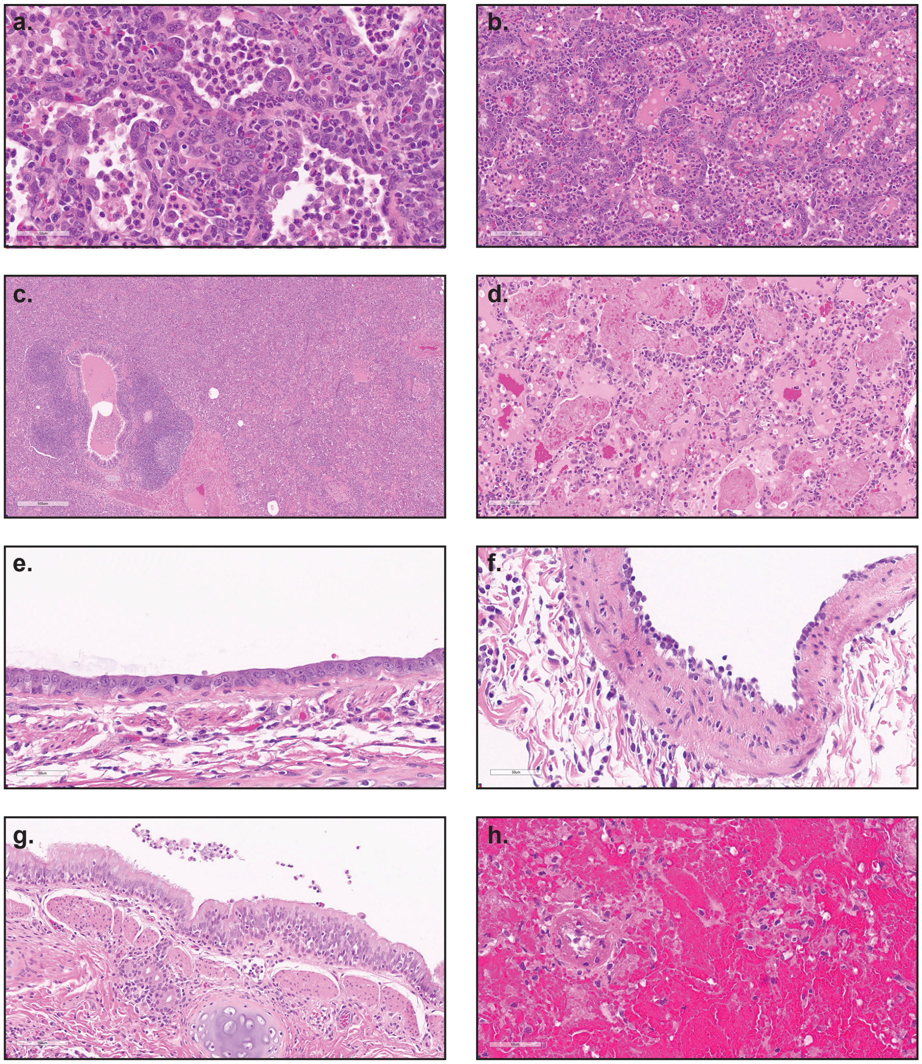

Supplementary Figure 5. Examples of types of lung pathology. Lung tissue sections were stained with hematoxylin and eosin (H&E). a) Prominent type-II pneumocyte hyperplasia, syncytia formation and alveoli filled with macrophages and neutrophils. Scale bar = 50 µm. b) Diffuse type-II pneumocyte hyperplasia and alveoli filled with neutrophils and macrophages admixed with protein-rich edema fluid. Scale bar = 100 µm. c) Bronchus-associated lymphoid hyperplasia with lymphofollicular formation. Bronchial lumen contains macrophages and neutrophils admixed with protein-rich fluid. Scale bar = 500 µm. d) Necrotizing hemorrhagic, fibrinous interstitial pneumonia with septal necrosis, and alveoli filled with dense mats of fibrin, hemorrhage and edema fluid. Scale bar = 100 µm. e) Bronchiole lined by attenuated epithelium with ciliary loss. Scale bar = 50µm. f) Intrapulmonary vessel exhibiting endothelial hypertrophy, rounding up of endothelial cells and disruption of endothelium with infiltrating leukocytes. Scale bar = 50 µm. g) Bronchus lined by hyperplastic epithelium with lymphocytic infiltration. Scale bar = 100 µm. h) Intrapulmonary artery with fibrinoid necrosis of vascular wall with severe hemorrhage and necrosis of surrounding tissue. Scale bar = 50 µm.

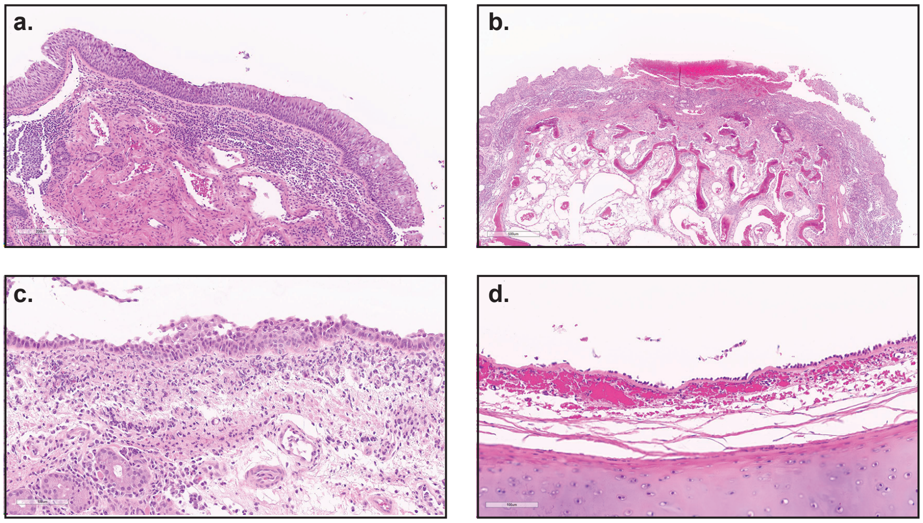

Supplementary Figure 6. Examples of upper airway and tracheal pathology. Tissue sections were stained with H&E. a) Nasal mucosa tissues with lymphocytic infiltration of epithelium, multifocal ciliary loss, moderate plasmacytic and lymphocytic inflammation in the lamina propria. Scale bar = 200 µm. b) Nasal mucosa with erosion and ulceration, luminal hemorrhage, luminal neutrophilic exudate. Scale bar = 500 µm. c) Trachea epithelial erosion and mixed inflammatory infiltrate (lymphocytes, plasma cells and macrophages) in the lamina propria. Scale bar = 100 µm. d) Trachea epithelial denudation with proprial hemorrhage, congestion and edema. Scale bar = 100 µm.

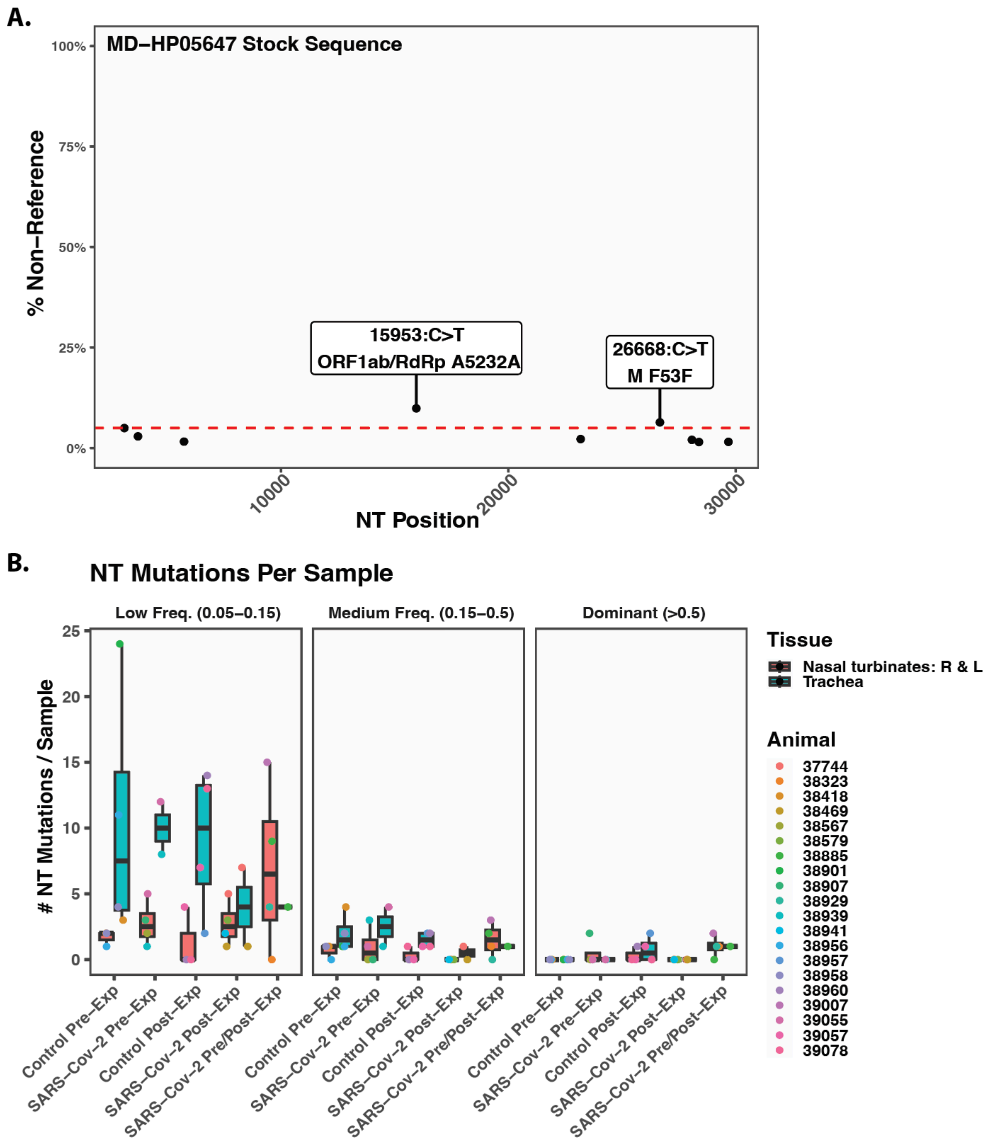

Supplementary Figure 7. Effect of Inhaled Aerosolized Anti-SARS-CoV-2 mAb Treatment on SARS-CoV-2 Evolution. (A) Viral RNA was isolated from the challenge stock, followed by an amplicon-based sequencing approach, high-depth Illumina sequencing and alignment to the reference sequence. The graph displays the fraction of non-reference bases detected at each genomic position. The dotted red line denotes 5% of total reads. A total of two nucleotide positions were detected with mutations in >5% of reads, which are labeled with the nucleotide and predicted amino acid changes. (B) Viral RNA was isolated from nasal turbinates and trachea tissues, followed by amplicon-based sequencing. The box plot summarizes the total number of nucleotide mutations detected in each sample, categorized by the frequency of each mutation. Very few medium frequency or dominant mutations were detected, with no significant differences between cohorts. The majority of samples contained low-frequency mutations. Key on the right shows a unique color that was assigned to each animal.

Supplementary Table 1. Deep Sequence Analysis of SARS-CoV-2 Delta in Nasal Turbinates and Trachea from Control mAb Pre-exposure Treatment Group

Cohort	Animal	Tissue	Start	Ref	AltAlleles	Depth	Freq	ORF	Protein	Amino Acid	Category
Control Pre-Exp	38418	Nasal turbinates: R & L	4449	C	T	302	6.0%	ORF1ab	GU280_gp01,nsp3	p.Ala1397Ala	Synonymous
Control Pre-Exp	38418	Nasal turbinates: R & L	15953	C	T	591	14.9%	ORF1ab	GU280_gp01,RNA-dependent_RNA_polymerase	p.Ala5232Ala	Synonymous
Control Pre-Exp	38418	Nasal turbinates: R & L	20714	A	G	125	31.2%	ORF1ab	2'-O-ribose_methyltransferase,GU280_gp01	p.Leu6819Leu	Synonymous
Control Pre-Exp	38418	Trachea	9586	T	C	507	27.6%	ORF1ab	GU280_gp01,nsp4	p.Leu3110Leu	Synonymous
Control Pre-Exp	38418	Trachea	9828	T	C	682	28.2%	ORF1ab	GU280_gp01,nsp4	p.Tyr3190Tyr	Synonymous
Control Pre-Exp	38418	Trachea	14315	C	T	409	18.3%	ORF1ab	GU280_gp01,RNA-dependent_RNA_polymerase	p.Tyr4686Tyr	Synonymous
Control Pre-Exp	38418	Trachea	21938	T	C	783	22.9%	S	GU280_gp02	p.Ile128Thr	Non-synonymous
Control Pre-Exp	38418	Trachea	22278	T	C	739	14.2%	S	GU280_gp02	p.Ala241Ala	Synonymous
Control Pre-Exp	38418	Trachea	24482	A	G	144	12.5%	S	GU280_gp02	p.Asn976Ser	Non-synonymous
Control Pre-Exp	38418	Trachea	29558	C	A	439	8.2%	ORF10	GU280_gp11	p.Phe7Leu	Non-synonymous
Control Pre-Exp	38901	Trachea	959	A	G	95	6.3%	ORF1ab	GU280_gp01,nsp2	p.His234Arg	Non-synonymous
Control Pre-Exp	38901	Trachea	3761	C	T	223	7.2%	ORF1ab	GU280_gp01,nsp3	p.Thr1168Ile	Non-synonymous
Control Pre-Exp	38901	Trachea	4103	A	T	171	6.4%	ORF1ab	GU280_gp01,nsp3	p.Asp1282Val	Non-synonymous
Control Pre-Exp	38901	Trachea	4743	A	G	162	11.7%	ORF1ab	GU280_gp01,nsp3	p.Lys1495Lys	Synonymous
Control Pre-Exp	38901	Trachea	5987	A	G	149	9.4%	ORF1ab	GU280_gp01,nsp3	p.Gln1910Arg	Non-synonymous
Control Pre-Exp	38901	Trachea	6518	C	T	124	6.5%	ORF1ab	GU280_gp01,nsp3	p.Thr2087Ile	Non-synonymous
Control Pre-Exp	38901	Trachea	7494	T	C	68	7.4%	ORF1ab	GU280_gp01,nsp3	p.Cys2412Cys	Synonymous
Control Pre-Exp	38901	Trachea	8151	T	C	86	8.1%	ORF1ab	GU280_gp01,nsp3	p.Ser2631Ser	Synonymous
Control Pre-Exp	38901	Trachea	8292	C	T	104	10.6%	ORF1ab	GU280_gp01,nsp3	p.Asn2678Asn	Synonymous
Control Pre-Exp	38901	Trachea	10615	A	G	656	8.8%	ORF1ab	3C-like_proteinase,GU280_gp01	p.Thr3453Ala	Non-synonymous
Control Pre-Exp	38901	Trachea	11164	C	T	349	9.7%	ORF1ab	GU280_gp01,nsp6	p.Leu3636Phe	Non-synonymous
Control Pre-Exp	38901	Trachea	11318	C	T	485	11.8%	ORF1ab	GU280_gp01,nsp6	p.Ser3687Leu	Non-synonymous
Control Pre-Exp	38901	Trachea	12095	C	T	333	6.9%	ORF1ab	GU280_gp01,nsp8	p.Ser3946Leu	Non-synonymous
Control Pre-Exp	38901	Trachea	13495	C	T	332	25.6%	ORF1ab	GU280_gp01,RNA-dependent_RNA_polymerase	p.Pro4413Leu	Non-synonymous
Control Pre-Exp	38901	Trachea	15447	T	C	54	7.4%	ORF1ab	GU280_gp01,RNA-dependent_RNA_polymerase	p.Ser5064Pro	Non-synonymous
Control Pre-Exp	38901	Trachea	15953	C	T	117	5.1%	ORF1ab	GU280_gp01,RNA-dependent_RNA_polymerase	p.Ala5232Ala	Synonymous
Control Pre-Exp	38901	Trachea	17315	A	G	112	5.4%	ORF1ab	GU280_gp01,helicase	p.Ala5686Ala	Synonymous
Control Pre-Exp	38901	Trachea	20687	T	C	95	7.4%	ORF1ab	2'-O-ribose_methyltransferase,GU280_gp01	p.Pro6810Pro	Synonymous
Control Pre-Exp	38901	Trachea	22263	T	C	395	8.4%	S	GU280_gp02	p.Phe236Phe	Synonymous
Control Pre-Exp	38901	Trachea	22485	C	T	230	9.1%	S	GU280_gp02	p.Ile310Ile	Synonymous
Control Pre-Exp	38901	Trachea	24155	T	C	156	5.1%	S	GU280_gp02	p.Met867Thr	Non-synonymous
Control Pre-Exp	38901	Trachea	24575	C	T	65	9.2%	S	GU280_gp02	p.Thr1007Ile	Non-synonymous
Control Pre-Exp	38901	Trachea	24803	G	A	196	8.2%	S	GU280_gp02	p.Gly1083Glu	Non-synonymous
Control Pre-Exp	38901	Trachea	25171	C	T	223	11.2%	S	GU280_gp02	p.Gln1206*	Non-synonymous
Control Pre-Exp	38901	Trachea	26668	C	T	286	9.1%	M	GU280_gp05	p.Phe53Phe	Synonymous
Control Pre-Exp	38956	Nasal turbinates: R & L	28367	A	C	1641	7.7%	N	GU280_gp10	p.Lys38Asn	Non-synonymous
Control Pre-Exp	38956	Trachea	71	T	A	76	7.9%	NON-CODING	NON-CODING		
Control Pre-Exp	38956	Trachea	1955	C	T	76	5.3%	ORF1ab	GU280_gp01,nsp2	p.Ala566Val	Non-synonymous
Control Pre-Exp	38956	Trachea	3565	T	G	164	6.7%	ORF1ab	GU280_gp01,nsp3	p.Cys1103Gly	Non-synonymous
Control Pre-Exp	38956	Trachea	3714	T	C	155	6.5%	ORF1ab	GU280_gp01,nsp3	p.Gly1152Gly	Synonymous
Control Pre-Exp	38956	Trachea	4318	T	C	196	11.7%	ORF1ab	GU280_gp01,nsp3	p.Tyr1354His	Non-synonymous
Control Pre-Exp	38956	Trachea	5173	G	A	149	5.4%	ORF1ab	GU280_gp01,nsp3	p.Asp1639Asn	Non-synonymous
Control Pre-Exp	38956	Trachea	13453	T	C	581	6.7%	ORF1ab	GU280_gp01,nsp11,RNA-dependent_RNA_polymerase	p.Phe4399Leu	Non-synonymous
Control Pre-Exp	38956	Trachea	15953	C	T	140	10.0%	ORF1ab	GU280_gp01,RNA-dependent_RNA_polymerase	p.Ala5232Ala	Synonymous
Control Pre-Exp	38956	Trachea	18130	T	C	542	5.5%	ORF1ab	3'-to-5'_exonuclease,GU280_gp01	p.Phe5958Ser	Non-synonymous
Control Pre-Exp	38956	Trachea	22060	G	C	353	5.1%	S	GU280_gp02	p.Val169Leu	Non-synonymous
Control Pre-Exp	38956	Trachea	23894	T	C	268	7.5%	S	GU280_gp02	p.Phe780Ser	Non-synonymous
Control Pre-Exp	38956	Trachea	26668	C	T	149	16.1%	M	GU280_gp05	p.Phe53Phe	Synonymous
Control Pre-Exp	38958	Nasal turbinates: R & L	5805	C	T	383	24.8%	ORF1ab	GU280_gp01,nsp3	p.Asp1849Asp	Synonymous
Control Pre-Exp	38958	Nasal turbinates: R & L	15953	C	T	824	6.9%	ORF1ab	GU280_gp01,RNA-dependent_RNA_polymerase	p.Ala5232Ala	Synonymous
Control Pre-Exp	38958	Nasal turbinates: R & L	19306	C	T	256	11.7%	ORF1ab	3'-to-5'_exonuclease,GU280_gp01	p.Ala6350Val	Non-synonymous
Control Pre-Exp	38958	Trachea	6099	T	C	279	8.2%	ORF1ab	GU280_gp01,nsp3	p.Tyr1947Tyr	Synonymous
Control Pre-Exp	38958	Trachea	6276	T	C	228	8.3%	ORF1ab	GU280_gp01,nsp3	p.Asn2006Asn	Synonymous
Control Pre-Exp	38958	Trachea	6746	G	A	107	5.6%	ORF1ab	GU280_gp01,nsp3	p.Arg2163His	Non-synonymous
Control Pre-Exp	38958	Trachea	20755	C	T	122	27.0%	ORF1ab	2'-O-ribose_methyltransferase,GU280_gp01	p.Thr6833Ile	Non-synonymous
Control Pre-Exp	38958	Trachea	23973	T	C	439	10.9%	S	GU280_gp02	p.Asp806Asp	Synonymous
Control Pre-Exp	38958	Trachea	26692	T	C	389	16.7%	M	GU280_gp05	p.Thr61Thr	Synonymous

Supplementary Table 2. Deep Sequence Analysis of SARS-CoV-2 Delta in Nasal Turbinates and Trachea from AR-701 mAb Pre-exposure Treatment Group

Cohort	Animal	Tissue	Start	Ref	AltAlleles	Depth	Freq	ORF	Protein	Amino Acid	Category
SARS-Cov-2 Pre-Exp	38567	Nasal turbinates: R & L	4449	C	T	247	12.1%	ORF1ab	GU280_gp01,nsp3	p.Ala1397Ala	Synonymous
SARS-Cov-2 Pre-Exp	38567	Nasal turbinates: R & L	28074	C	T	1128	10.6%	ORF8	GU280_gp09	p.Ala65Val	Non-synonymous
SARS-Cov-2 Pre-Exp	38907	Nasal turbinates: R & L	5232	C	T	257	80.9%	ORF1ab	GU280_gp01,nsp3	p.Tyr1658Tyr	Synonymous
SARS-Cov-2 Pre-Exp	38907	Nasal turbinates: R & L	15755	T	C	916	5.2%	ORF1ab	GU280_gp01,RNA-dependent_RNA_polymerase	p.Gly5166Gly	Synonymous
SARS-Cov-2 Pre-Exp	38907	Nasal turbinates: R & L	15953	C	T	1342	81.1%	ORF1ab	GU280_gp01,RNA-dependent_RNA_polymerase	p.Ala5232Ala	Synonymous
SARS-Cov-2 Pre-Exp	38907	Nasal turbinates: R & L	26668	C	T	651	14.9%	M	GU280_gp05	p.Phe53Phe	Synonymous
SARS-Cov-2 Pre-Exp	38907	Nasal turbinates: R & L	29607	C	T	684	7.2%	ORF10	GU280_gp11	p.Arg24Cys	Non-synonymous
SARS-Cov-2 Pre-Exp	38939	Nasal turbinates: R & L	3048	T	C	247	18.2%	ORF1ab	GU280_gp01,nsp3	p.Asp930Asp	Synonymous
SARS-Cov-2 Pre-Exp	38939	Nasal turbinates: R & L	11332	C	T	1031	5.0%	ORF1ab	GU280_gp01,nsp6	p.Leu3692Leu	Synonymous
SARS-Cov-2 Pre-Exp	38939	Nasal turbinates: R & L	17390	C	T	343	16.9%	ORF1ab	GU280_gp01,helicase	p.Val5711Val	Synonymous
SARS-Cov-2 Pre-Exp	38939	Nasal turbinates: R & L	28074	C	T	2095	22.2%	ORF8	GU280_gp09	p.Ala65Val	Non-synonymous
SARS-Cov-2 Pre-Exp	38939	Trachea	3254	C	T	350	5.1%	ORF1ab	GU280_gp01,nsp3	p.Thr999Ile	Non-synonymous
SARS-Cov-2 Pre-Exp	38939	Trachea	3582	C	T	842	6.2%	ORF1ab	GU280_gp01,nsp3	p.His1108His	Synonymous
SARS-Cov-2 Pre-Exp	38939	Trachea	3710	C	A	700	13.9%	ORF1ab	GU280_gp01,nsp3	p.Ala1151Asp	Non-synonymous
SARS-Cov-2 Pre-Exp	38939	Trachea	4378	A	C	444	7.7%	ORF1ab	GU280_gp01,nsp3	p.Asn1374His	Non-synonymous
SARS-Cov-2 Pre-Exp	38939	Trachea	11332	C	T	861	35.8%	ORF1ab	GU280_gp01,nsp6	p.Leu3692Leu	Synonymous
SARS-Cov-2 Pre-Exp	38939	Trachea	12225	T	A	825	5.3%	ORF1ab	GU280_gp01,nsp8	p.Ser3989Ser	Synonymous
SARS-Cov-2 Pre-Exp	38939	Trachea	15000	G	C	229	10.5%	ORF1ab	GU280_gp01,RNA-dependent_RNA_polymerase	p.Asp4915His	Non-synonymous
SARS-Cov-2 Pre-Exp	38939	Trachea	20027	T	A	84	7.1%	ORF1ab	endoRNAse,GU280_gp01	p.Arg6590Arg	Synonymous
SARS-Cov-2 Pre-Exp	38939	Trachea	29525	C	T	1386	7.1%	N-ORF10	GU280_gp10-GU280_gp11		
SARS-Cov-2 Pre-Exp	39055	Nasal turbinates: R & L	586	C	T	596	7.7%	ORF1ab	GU280_gp01,leader_protein	p.His110Tyr	Non-synonymous
SARS-Cov-2 Pre-Exp	39055	Nasal turbinates: R & L	3102	G	T	209	9.1%	ORF1ab	GU280_gp01,nsp3	p.Glu948Asp	Non-synonymous
SARS-Cov-2 Pre-Exp	39055	Nasal turbinates: R & L	4267	T	C	292	6.5%	ORF1ab	GU280_gp01,nsp3	p.Tyr1337His	Non-synonymous
SARS-Cov-2 Pre-Exp	39055	Nasal turbinates: R & L	6098	A	G	420	11.9%	ORF1ab	GU280_gp01,nsp3	p.Tyr1947Cys	Non-synonymous
SARS-Cov-2 Pre-Exp	39055	Nasal turbinates: R & L	7827	C	T	410	6.1%	ORF1ab	GU280_gp01,nsp3	p.Asn2523Asn	Synonymous
SARS-Cov-2 Pre-Exp	39055	Nasal turbinates: R & L	15953	C	T	835	15.6%	ORF1ab	GU280_gp01,RNA-dependent_RNA_polymerase	p.Ala5232Ala	Synonymous
SARS-Cov-2 Pre-Exp	39055	Trachea	3532	G	A	579	5.5%	ORF1ab	GU280_gp01,nsp3	p.Ala1092Thr	Non-synonymous
SARS-Cov-2 Pre-Exp	39055	Trachea	5137	C	T	124	26.6%	ORF1ab	GU280_gp01,nsp3	p.Leu1627Leu	Synonymous
SARS-Cov-2 Pre-Exp	39055	Trachea	7790	A	G	261	12.3%	ORF1ab	GU280_gp01,nsp3	p.Lys2511Arg	Non-synonymous
SARS-Cov-2 Pre-Exp	39055	Trachea	9662	T	C	851	6.2%	ORF1ab	GU280_gp01,nsp4	p.Leu3135Ser	Non-synonymous
SARS-Cov-2 Pre-Exp	39055	Trachea	11697	C	T	451	8.2%	ORF1ab	GU280_gp01,nsp6	p.Tyr3813Tyr	Synonymous
SARS-Cov-2 Pre-Exp	39055	Trachea	12248	T	C	758	7.4%	ORF1ab	GU280_gp01,nsp8	p.Met3997Thr	Non-synonymous
SARS-Cov-2 Pre-Exp	39055	Trachea	12486	C	T	537	16.4%	ORF1ab	GU280_gp01,nsp8	p.Asp4076Asp	Synonymous
SARS-Cov-2 Pre-Exp	39055	Trachea	14030	T	A	1175	5.2%	ORF1ab	GU280_gp01,RNA-dependent_RNA_polymerase	p.Ala4591Ala	Synonymous
SARS-Cov-2 Pre-Exp	39055	Trachea	16995	C	G	74	20.3%	ORF1ab	GU280_gp01,helicase	p.Leu5580Val	Non-synonymous
SARS-Cov-2 Pre-Exp	39055	Trachea	17789	T	C	672	6.3%	ORF1ab	GU280_gp01,helicase	p.Ala5844Ala	Synonymous
SARS-Cov-2 Pre-Exp	39055	Trachea	19285	G	T	725	6.5%	ORF1ab	3'-to-5'_exonuclease,GU280_gp01	p.Ser6343Ile	Non-synonymous
SARS-Cov-2 Pre-Exp	39055	Trachea	19323	T	C	603	10.6%	ORF1ab	3'-to-5'_exonuclease,GU280_gp01	p.Phe6356Leu	Non-synonymous
SARS-Cov-2 Pre-Exp	39055	Trachea	22754	T	C	695	8.8%	S	GU280_gp02	p.Ile400Thr	Non-synonymous
SARS-Cov-2 Pre-Exp	39055	Trachea	24845	G	T	211	13.7%	S	GU280_gp02	p.Gly1097Val	Non-synonymous
SARS-Cov-2 Pre-Exp	39055	Trachea	25087	C	A	203	28.6%	S	GU280_gp02	p.Gln1178Lys	Non-synonymous
SARS-Cov-2 Pre-Exp	39055	Trachea	26635	G	T	396	7.3%	M	GU280_gp05	p.Arg42Ser	Non-synonymous

Supplementary Table 3. Deep Sequence Analysis of SARS-CoV-2 Delta in Nasal Turbinates and Trachea from Control mAb Post-exposure Treatment Group

Cohort	Animal	Tissue	Start	Ref	AltAlleles	Depth	Freq	ORF	Protein	Amino Acid	Category
Control Post-Exp	38957	Trachea	619	G	A	110	27.3%	ORF1ab	GU280_gp01,leader_protein	p.Val121Ile	Non-synonymous
Control Post-Exp	38957	Trachea	11060	C	T	519	40.5%	ORF1ab	GU280_gp01,nsp6	p.Ser3601Phe	Non-synonymous
Control Post-Exp	38957	Trachea	24736	T	C	128	97.7%	S	GU280_gp02	p.Leu1061Leu	Synonymous
Control Post-Exp	38957	Trachea	24910	T	C	111	100.0%	S	GU280_gp02	p.Phe1119Leu	Non-synonymous
Control Post-Exp	38957	Trachea	28317	G	T	2645	8.7%	N	GU280_gp10	p.Asp22Tyr	Non-synonymous
Control Post-Exp	38957	Trachea	28715	T	C	2593	9.2%	N	GU280_gp10	p.Asn154Asn	Synonymous
Control Post-Exp	38960	Nasal turbinates: R & L	10108	A	G	128	50.0%	ORF1ab	3C-like_proteinase,GU280_gp01	p.Thr3284Ala	Non-synonymous
Control Post-Exp	38960	Trachea	172	T	C	75	12.0%	NON-CODING	NON-CODING		
Control Post-Exp	38960	Trachea	1953	C	A	90	5.6%	ORF1ab	GU280_gp01,nsp2	p.Ala565Ala	Synonymous
Control Post-Exp	38960	Trachea	3914	A	G	68	11.8%	ORF1ab	GU280_gp01,nsp3	p.Lys1219Arg	Non-synonymous
Control Post-Exp	38960	Trachea	4167	T	A	82	6.1%	ORF1ab	GU280_gp01,nsp3	p.Thr1303Thr	Synonymous
Control Post-Exp	38960	Trachea	4875	T	C	130	26.2%	ORF1ab	GU280_gp01,nsp3	p.Ser1539Ser	Synonymous
Control Post-Exp	38960	Trachea	7727	T	G	143	6.3%	ORF1ab	GU280_gp01,nsp3	p.Ile2490Ser	Non-synonymous
Control Post-Exp	38960	Trachea	9934	T	G	1035	8.3%	ORF1ab	GU280_gp01,nsp4	p.Tyr3226Asp	Non-synonymous
Control Post-Exp	38960	Trachea	14886	G	A	76	7.9%	ORF1ab	GU280_gp01,RNA-dependent_RNA_polymerase	p.Gly4877Ser	Non-synonymous
Control Post-Exp	38960	Trachea	17113	T	C	78	9.0%	ORF1ab	GU280_gp01,helicase	p.Leu5619Pro	Non-synonymous
Control Post-Exp	38960	Trachea	18479	C	A	317	6.3%	ORF1ab	3'-to-5'_exonuclease,GU280_gp01	p.Leu6074Leu	Synonymous
Control Post-Exp	38960	Trachea	18972	C	T	839	28.1%	ORF1ab	3'-to-5'_exonuclease,GU280_gp01	p.His6239Tyr	Non-synonymous
Control Post-Exp	38960	Trachea	19178	C	T	571	10.2%	ORF1ab	3'-to-5'_exonuclease,GU280_gp01	p.Cys6307Cys	Synonymous
Control Post-Exp	38960	Trachea	22211	C	T	802	8.6%	S	GU280_gp02	p.Ser219Leu	Non-synonymous
Control Post-Exp	38960	Trachea	22602	T	C	292	11.3%	S	GU280_gp02	p.Tyr349Tyr	Synonymous
Control Post-Exp	38960	Trachea	23269	G	T	691	13.7%	S	GU280_gp02	p.Asp572Tyr	Non-synonymous
Control Post-Exp	38960	Trachea	24385	G	A	260	6.5%	S	GU280_gp02	p.Gly944Arg	Non-synonymous
Control Post-Exp	39057	Nasal turbinates: R & L	396	T	A	112	8.9%	ORF1ab	GU280_gp01,leader_protein	p.Leu46Leu	Synonymous
Control Post-Exp	39057	Nasal turbinates: R & L	4449	C	T	205	5.9%	ORF1ab	GU280_gp01,nsp3	p.Ala1397Ala	Synonymous
Control Post-Exp	39057	Nasal turbinates: R & L	15953	C	T	615	10.9%	ORF1ab	GU280_gp01,RNA-dependent_RNA_polymerase	p.Ala5232Ala	Synonymous
Control Post-Exp	39057	Nasal turbinates: R & L	24850	C	T	127	5.5%	S	GU280_gp02	p.His1099Tyr	Non-synonymous
Control Post-Exp	39057	Trachea	59	C	T	52	7.7%	NON-CODING	NON-CODING		
Control Post-Exp	39057	Trachea	4474	T	A	320	6.3%	ORF1ab	GU280_gp01,nsp3	p.Tyr1406Asn	Non-synonymous
Control Post-Exp	39057	Trachea	4692	T	C	289	5.2%	ORF1ab	GU280_gp01,nsp3	p.Ser1478Ser	Synonymous
Control Post-Exp	39057	Trachea	9231	A	T	66	7.6%	ORF1ab	GU280_gp01,nsp4	p.Arg2991Ser	Non-synonymous
Control Post-Exp	39057	Trachea	17297	C	T	161	8.7%	ORF1ab	GU280_gp01,helicase	p.Val5680Val	Synonymous
Control Post-Exp	39057	Trachea	18884	G	T	979	7.9%	ORF1ab	3'-to-5'_exonuclease,GU280_gp01	p.Glu6209Asp	Non-synonymous
Control Post-Exp	39057	Trachea	21320	C	A	132	5.3%	ORF1ab	2'-O-ribose_methyltransferase,GU280_gp01	p.Val7021Val	Synonymous
Control Post-Exp	39057	Trachea	26905	T	C	96	67.7%	M	GU280_gp05	p.Pro132Pro	Synonymous
Control Post-Exp	39057	Trachea	29305	T	A	656	23.2%	N	GU280_gp10	p.Ile351Asn	Non-synonymous
Control Post-Exp	39078	Nasal turbinates: R & L	15953	C	T	1070	22.1%	ORF1ab	GU280_gp01,RNA-dependent_RNA_polymerase	p.Ala5232Ala	Synonymous
Control Post-Exp	39078	Trachea	3276	T	A	251	5.2%	ORF1ab	GU280_gp01,nsp3	p.Val1006Val	Synonymous
Control Post-Exp	39078	Trachea	4808	C	T	301	6.6%	ORF1ab	GU280_gp01,nsp3	p.Ser1517Phe	Non-synonymous
Control Post-Exp	39078	Trachea	5232	C	T	98	6.1%	ORF1ab	GU280_gp01,nsp3	p.Tyr1658Tyr	Synonymous
Control Post-Exp	39078	Trachea	5283	T	C	434	12.7%	ORF1ab	GU280_gp01,nsp3	p.Tyr1675Tyr	Synonymous
Control Post-Exp	39078	Trachea	7150	T	C	53	20.8%	ORF1ab	GU280_gp01,nsp3	p.Leu2298Leu	Synonymous
Control Post-Exp	39078	Trachea	7413	C	T	122	7.4%	ORF1ab	GU280_gp01,nsp3	p.Ile2385Ile	Synonymous
Control Post-Exp	39078	Trachea	11068	T	C	836	7.2%	ORF1ab	GU280_gp01,nsp6	p.Phe3604Leu	Non-synonymous
Control Post-Exp	39078	Trachea	13252	T	C	813	8.0%	ORF1ab	GU280_gp01,nsp10	p.Cys4332Arg	Non-synonymous
Control Post-Exp	39078	Trachea	15953	C	T	304	9.2%	ORF1ab	GU280_gp01,RNA-dependent_RNA_polymerase	p.Ala5232Ala	Synonymous
Control Post-Exp	39078	Trachea	17062	C	A	168	11.9%	ORF1ab	GU280_gp01,helicase	p.Ser5602Tyr	Non-synonymous
Control Post-Exp	39078	Trachea	21568	C	T	297	7.1%	S	GU280_gp02	p.Leu5Phe	Non-synonymous
Control Post-Exp	39078	Trachea	25461	T	C	286	6.3%	ORF3a	GU280_gp03	p.Phe28Leu	Non-synonymous
Control Post-Exp	39078	Trachea	26816	T	G	288	7.6%	M	GU280_gp05	p.Phe103Val	Non-synonymous
Control Post-Exp	39078	Trachea	28074	C	T	2208	7.2%	ORF8	GU280_gp09	p.Ala65Val	Non-synonymous

Supplementary Table 4. Deep Sequence Analysis of SARS-CoV-2 Delta in Nasal Turbinates and Trachea from AR-701 mAb Post-exposure Treatment Group

Cohort	Animal	Tissue	Start	Ref	AltAlleles	Depth	Freq	ORF	Protein	Amino Acid	Category
SARS-Cov-2 Post-Exp	37744	Nasal turbinates: R & L	5819	C	T	266	13.5%	ORF1ab	GU280_gp01,nsp3	p.Thr1854Ile	Non-synonymous
SARS-Cov-2 Post-Exp	37744	Nasal turbinates: R & L	10226	G	T	783	7.3%	ORF1ab	3C-like_proteinase,GU280_gp01	p.Arg3323Leu	Non-synonymous
SARS-Cov-2 Post-Exp	37744	Nasal turbinates: R & L	15953	C	T	348	13.8%	ORF1ab	GU280_gp01,RNA-dependent_RNA_polymerase	p.Ala5232Ala	Synonymous
SARS-Cov-2 Post-Exp	37744	Nasal turbinates: R & L	25748	A	T	814	5.5%	ORF3a	GU280_gp03	p.Ile123Ile	Synonymous
SARS-Cov-2 Post-Exp	37744	Nasal turbinates: R & L	26354	G	A	759	5.0%	E	GU280_gp04	p.Ala41Ala	Synonymous
SARS-Cov-2 Post-Exp	37744	Trachea	4174	T	G	153	7.8%	ORF1ab	GU280_gp01,nsp3	p.Ser1306Ala	Non-synonymous
SARS-Cov-2 Post-Exp	37744	Trachea	4359	T	C	377	9.0%	ORF1ab	GU280_gp01,nsp3	p.Ile1367Ile	Synonymous
SARS-Cov-2 Post-Exp	37744	Trachea	9672	C	T	1100	8.8%	ORF1ab	GU280_gp01,nsp4	p.Phe3138Phe	Synonymous
SARS-Cov-2 Post-Exp	37744	Trachea	11068	T	C	548	7.3%	ORF1ab	GU280_gp01,nsp6	p.Phe3604Leu	Non-synonymous
SARS-Cov-2 Post-Exp	37744	Trachea	17003	C	T	335	31.9%	ORF1ab	GU280_gp01,helicase	p.Ile5582Ile	Synonymous
SARS-Cov-2 Post-Exp	37744	Trachea	17538	T	C	263	8.0%	ORF1ab	GU280_gp01,helicase	p.Phe5761Leu	Non-synonymous
SARS-Cov-2 Post-Exp	37744	Trachea	19825	T	C	219	6.4%	ORF1ab	endoRNAse,GU280_gp01	p.Ile6523Thr	Non-synonymous
SARS-Cov-2 Post-Exp	37744	Trachea	22354	T	C	531	6.8%	S	GU280_gp02	p.Tyr267His	Non-synonymous
SARS-Cov-2 Post-Exp	38469	Nasal turbinates: R & L	8347	C	G	67	13.4%	ORF1ab	GU280_gp01,nsp3	p.Arg2697Gly	Non-synonymous
SARS-Cov-2 Post-Exp	38469	Trachea	24909	A	T	176	5.1%	S	GU280_gp02	p.Thr1118Thr	Synonymous
SARS-Cov-2 Post-Exp	38579	Nasal turbinates: R & L	3995	C	T	259	5.0%	ORF1ab	GU280_gp01,nsp3	p.Thr1246Ile	Non-synonymous
SARS-Cov-2 Post-Exp	38579	Nasal turbinates: R & L	7011	T	G	91	5.5%	ORF1ab	GU280_gp01,nsp3	p.Gly2251Gly	Synonymous
SARS-Cov-2 Post-Exp	38579	Nasal turbinates: R & L	26525	G	T	445	12.4%	M	GU280_gp05	p.Gly6Cys	Non-synonymous
SARS-Cov-2 Post-Exp	38941	Nasal turbinates: R & L	15953	C	T	795	8.7%	ORF1ab	GU280_gp01,RNA-dependent_RNA_polymerase	p.Ala5232Ala	Synonymous
SARS-Cov-2 Post-Exp	38941	Nasal turbinates: R & L	23628	T	C	721	5.8%	S	GU280_gp02	p.Ile691Ile	Synonymous

Supplementary Table 5. Deep Sequence Analysis of SARS-CoV-2 Delta in Nasal Turbinates and Trachea from AR-701 mAb Pre/Post-exposure Treatment Group

Cohort	Animal	Tissue	Start	Ref	AltAlleles	Depth	Freq	ORF	Protein	Amino Acid	Category
SARS-Cov-2 Pre/Post-Exp	38323	Nasal turbinates: R & L	19010	C	T	419	58.5%	ORF1ab	3'-to-5'_exonuclease,GU280_gp01	p.Phe6251Phe	Synonymous
SARS-Cov-2 Pre/Post-Exp	38323	Nasal turbinates: R & L	22973	C	A	217	27.2%	S	GU280_gp02	p.Ala473Asp	Non-synonymous
SARS-Cov-2 Pre/Post-Exp	38885	Nasal turbinates: R & L	376	T	C	254	5.1%	ORF1ab	GU280_gp01,leader_protein	p.Ser40Pro	Non-synonymous
SARS-Cov-2 Pre/Post-Exp	38885	Nasal turbinates: R & L	2008	A	G	136	8.8%	ORF1ab	GU280_gp01,nsp2	p.Met584Val	Non-synonymous
SARS-Cov-2 Pre/Post-Exp	38885	Nasal turbinates: R & L	3376	A	T	243	5.8%	ORF1ab	GU280_gp01,nsp3	p.Ile1040Phe	Non-synonymous
SARS-Cov-2 Pre/Post-Exp	38885	Nasal turbinates: R & L	3955	A	G	155	6.5%	ORF1ab	GU280_gp01,nsp3	p.Lys1233Glu	Non-synonymous
SARS-Cov-2 Pre/Post-Exp	38885	Nasal turbinates: R & L	13465	T	G	521	5.4%	ORF1ab	GU280_gp01,nsp11,RNA-dependent_RNA_polymerase	p.Val4403Gly	Non-synonymous
SARS-Cov-2 Pre/Post-Exp	38885	Nasal turbinates: R & L	15662	T	C	63	7.9%	ORF1ab	GU280_gp01,RNA-dependent_RNA_polymerase	p.Asn5135Asn	Synonymous
SARS-Cov-2 Pre/Post-Exp	38885	Nasal turbinates: R & L	17345	C	T	67	22.4%	ORF1ab	GU280_gp01,helicase	p.Val5696Val	Synonymous
SARS-Cov-2 Pre/Post-Exp	38885	Nasal turbinates: R & L	18879	C	A	867	5.7%	ORF1ab	3'-to-5'_exonuclease,GU280_gp01	p.His6208Asn	Non-synonymous
SARS-Cov-2 Pre/Post-Exp	38885	Nasal turbinates: R & L	21753	T	C	623	5.9%	S	GU280_gp02	p.His66His	Synonymous
SARS-Cov-2 Pre/Post-Exp	38885	Nasal turbinates: R & L	24336	T	C	171	7.0%	S	GU280_gp02	p.Ser927Ser	Synonymous
SARS-Cov-2 Pre/Post-Exp	38885	Nasal turbinates: R & L	26026	C	T	786	24.7%	ORF3a	GU280_gp03	p.Ser216Leu	Non-synonymous
SARS-Cov-2 Pre/Post-Exp	38885	Trachea	4174	T	G	131	6.1%	ORF1ab	GU280_gp01,nsp3	p.Ser1306Ala	Non-synonymous
SARS-Cov-2 Pre/Post-Exp	38885	Trachea	7401	T	C	139	46.8%	ORF1ab	GU280_gp01,nsp3	p.Val2381Val	Synonymous
SARS-Cov-2 Pre/Post-Exp	38885	Trachea	15953	C	T	154	59.1%	ORF1ab	GU280_gp01,RNA-dependent_RNA_polymerase	p.Ala5232Ala	Synonymous
SARS-Cov-2 Pre/Post-Exp	38885	Trachea	16422	T	G	146	8.9%	ORF1ab	GU280_gp01,helicase	p.Leu5389Val	Non-synonymous
SARS-Cov-2 Pre/Post-Exp	38885	Trachea	19414	C	T	562	5.7%	ORF1ab	3'-to-5'_exonuclease,GU280_gp01	p.Ser6386Leu	Non-synonymous
SARS-Cov-2 Pre/Post-Exp	38885	Trachea	21098	T	C	157	6.4%	ORF1ab	2'-O-ribose_methyltransferase,GU280_gp01	p.Phe6947Phe	Synonymous
SARS-Cov-2 Pre/Post-Exp	38929	Nasal turbinates: R & L	10996	C	T	1399	5.8%	ORF1ab	GU280_gp01,nsp6	p.His3580Tyr	Non-synonymous
SARS-Cov-2 Pre/Post-Exp	38929	Nasal turbinates: R & L	24480	A	G	936	9.4%	S	GU280_gp02	p.Leu975Leu	Synonymous
SARS-Cov-2 Pre/Post-Exp	38929	Nasal turbinates: R & L	24575	C	T	731	97.9%	S	GU280_gp02	p.Thr1007Ile	Non-synonymous
SARS-Cov-2 Pre/Post-Exp	38929	Nasal turbinates: R & L	24668	T	G	653	5.1%	S	GU280_gp02	p.Val1038Gly	Non-synonymous
SARS-Cov-2 Pre/Post-Exp	38929	Nasal turbinates: R & L	24932	T	A	148	6.1%	S	GU280_gp02	p.Val1126Asp	Non-synonymous
SARS-Cov-2 Pre/Post-Exp	39007	Nasal turbinates: R & L	822	C	T	171	99.4%	ORF1ab	GU280_gp01,nsp2	p.Asn188Asn	Synonymous
SARS-Cov-2 Pre/Post-Exp	39007	Nasal turbinates: R & L	4034	T	G	212	11.3%	ORF1ab	GU280_gp01,nsp3	p.Ile1259Ser	Non-synonymous
SARS-Cov-2 Pre/Post-Exp	39007	Nasal turbinates: R & L	4923	T	C	68	26.5%	ORF1ab	GU280_gp01,nsp3	p.Asn1555Asn	Synonymous
SARS-Cov-2 Pre/Post-Exp	39007	Nasal turbinates: R & L	7406	T	G	104	9.6%	ORF1ab	GU280_gp01,nsp3	p.Met2383Arg	Non-synonymous
SARS-Cov-2 Pre/Post-Exp	39007	Nasal turbinates: R & L	9509	T	C	471	5.7%	ORF1ab	GU280_gp01,nsp4	p.Leu3084Pro	Non-synonymous
SARS-Cov-2 Pre/Post-Exp	39007	Nasal turbinates: R & L	10041	T	C	1499	6.1%	ORF1ab	GU280_gp01,nsp4	p.Val3261Val	Synonymous
SARS-Cov-2 Pre/Post-Exp	39007	Nasal turbinates: R & L	11302	T	A	1059	5.3%	ORF1ab	GU280_gp01,nsp6	p.Cys3682Ser	Non-synonymous
SARS-Cov-2 Pre/Post-Exp	39007	Nasal turbinates: R & L	12222	A	G	639	7.5%	ORF1ab	GU280_gp01,nsp8	p.Lys3988Lys	Synonymous
SARS-Cov-2 Pre/Post-Exp	39007	Nasal turbinates: R & L	12441	A	G	459	8.1%	ORF1ab	GU280_gp01,nsp8	p.Ile4061Met	Non-synonymous
SARS-Cov-2 Pre/Post-Exp	39007	Nasal turbinates: R & L	12518	C	T	416	98.8%	ORF1ab	GU280_gp01,nsp8	p.Thr4087Ile	Non-synonymous
SARS-Cov-2 Pre/Post-Exp	39007	Nasal turbinates: R & L	13853	C	T	783	5.1%	ORF1ab	GU280_gp01,RNA-dependent_RNA_polymerase	p.Asp4532Asp	Synonymous
SARS-Cov-2 Pre/Post-Exp	39007	Nasal turbinates: R & L	15282	T	C	134	9.0%	ORF1ab	GU280_gp01,RNA-dependent_RNA_polymerase	p.Trp5009Arg	Non-synonymous
SARS-Cov-2 Pre/Post-Exp	39007	Nasal turbinates: R & L	15799	A	G	95	6.3%	ORF1ab	GU280_gp01,RNA-dependent_RNA_polymerase	p.Gln5181Arg	Non-synonymous
SARS-Cov-2 Pre/Post-Exp	39007	Nasal turbinates: R & L	16180	G	T	194	5.2%	ORF1ab	GU280_gp01,RNA-dependent_RNA_polymerase	p.Trp5308Leu	Non-synonymous
SARS-Cov-2 Pre/Post-Exp	39007	Nasal turbinates: R & L	19304	T	C	520	12.9%	ORF1ab	3'-to-5'_exonuclease,GU280_gp01	p.His6349His	Synonymous
SARS-Cov-2 Pre/Post-Exp	39007	Nasal turbinates: R & L	21042	A	G	475	12.8%	ORF1ab	2'-O-ribose_methyltransferase,GU280_gp01	p.Met6929Val	Non-synonymous
SARS-Cov-2 Pre/Post-Exp	39007	Nasal turbinates: R & L	21328	C	A	220	17.3%	ORF1ab	2'-O-ribose_methyltransferase,GU280_gp01	p.Ala7024Glu	Non-synonymous
SARS-Cov-2 Pre/Post-Exp	39007	Nasal turbinates: R & L	22803	T	C	107	16.8%	S	GU280_gp02	p.Ile416Ile	Synonymous
SARS-Cov-2 Pre/Post-Exp	39007	Nasal turbinates: R & L	24830	T	C	116	10.3%	S	GU280_gp02	p.Val1092Ala	Non-synonymous
SARS-Cov-2 Pre/Post-Exp	39007	Nasal turbinates: R & L	25630	T	C	209	12.9%	ORF3a	GU280_gp03	p.Leu84Pro	Non-synonymous

Supplementary Table 6. ANOVA Dunnett's One-Sided Multiple Comparisons Statistical Analysis of Lung Pathology

Treatment Group	Mean Difference	95% CI of Difference	Adjusted P Value	Significance Difference Among Means (P<0.05)
Controls (n=8) vs. SARS-CoV-2 Pre-exposure (n=4)	7.988	2.269 to 13.71	0.0059	Yes
Controls (n=8) vs. SARS-CoV-2 Post-exposure (n=4)	7.125	1.406 to 12.84	0.0135	Yes
Controls (n=8) vs. SARS-CoV-2 Pre/Post- exposure (n=4)	7.063	1.344 to 12.78	0.143	No

Supplementary Table 7. Area Under the Curve (AUC) Tukey One-Way ANOVA Statistical Analysis of Cytokines in BAL samples

	**Controls (n=8)**		**SARS-CoV-2 Pre-Exposure (n=4)**			**SARS-CoV-2 Pre/Post-Exposure (n=4)**			**SARS-CoV-2 Post-Exposure (n=4)**		
	Mean	SEM	Mean	SEM	P	Mean	SEM	P	Mean	SEM	P
**CCL2**	2095.3	254.7	852.7	246.6	**0.088**	683.2	130.1	**0.048**	8891	1052.4	**<0.0001**
**CCL11**	222.8	77.6	7.2	1.7	0.112	10.7	4.8	0.119	134.9	77.3	0.731
**CXCL8**	283.6	100.2	29.3	12.6	0.131	9.3	3.8	0.097	343.6	65.7	0.935
**CXCL9**	198.7	26.3	13.5	3.9	**0.028**	11.7	4.9	**0.027**	484.1	105.6	**0.001**
**CXCL10**	70.3	9.4	27.64	6.5	0.593	22.4	3.5	0.506	414.9	93.9	**<0.0001**
**CXCL11**	2300.2	439.8	179.9	33.0	**0.010**	107.5	14.8	**0.008**	860.8	686.7	0.091
**CXCL13**	2684.7	516.3	268.8	46.0	**0.027**	255.6	33.5	**0.265**	2416.8	904.7	0.985
**G-CSF**	848.8	135.3	267.8	46.2	**0.008**	396.0	36.5	**0.040**	331.4	92.8	**0.018**
**IL-1β**	18.9	3.6	1.1	0.02	**0.004**	2.5	1.0	**0.008**	6.2	3.9	**0.042**
**IL-1RA**	8731.8	2486.8	868.8	111.3	**0.039**	1545	133.3	0.062	2621	545.4	0.127
**IL-6**	323.3	104.6	3.7	0.4	**0.047**	29.4	21.7	0.072	31.7	16.1	0.075
**IFN-α**	155.2	38.8	0.7	0	**0.029**	0.7	0	**0.029**	73.5	57.6	0.346
**TNF-α**	10.3	3.5	1.225	0	0.108	1.429	0.2	0.118	4.0	1.2	0.333

References

1. Van Rompay, K.K.A., *et al.* Early post-infection treatment of SARS-CoV-2 infected macaques with human convalescent plasma with high neutralizing activity had no antiviral effects but moderately reduced lung inflammation. *PLoS Pathog* **18**, e1009925 (2022).
