# Supplementary material for: Aerosol delivery of SARS-CoV-2 human monoclonal antibodies in macaques limits viral replication and lung pathology
Source: Nat Commun. 2023 Nov 3;14:7062. doi: 10.1038/s41467-023-42440-x (PMC10624670; doi:10.1038/s41467-023-42440-x)
Supplement: Supplementary file 3 — Reporting Summary [file 41467_2023_42440_MOESM3_ESM.pdf]

nature portfolio

Corresponding author(s): Nancy Haigwood for Vu Truong, Donald N. Forthal and Nancy L. Haigwood

Last updated by author(s): Oct 4, 2023

Reporting Summary

Nature Portfolio wishes to improve the reproducibility of the work that we publish. This form provides structure for consistency and transparency in reporting. For further information on Nature Portfolio policies, see our Editorial Policies and the Editorial Policy Checklist.

Statistics

For all statistical analyses, confirm that the following items are present in the figure legend, table legend, main text, or Methods section.

n/a	Confirmed	
☐	☒	The exact sample size (*n*) for each experimental group/condition, given as a discrete number and unit of measurement
☐	☒	A statement on whether measurements were taken from distinct samples or whether the same sample was measured repeatedly
☐	☒	The statistical test(s) used AND whether they are one- or two-sided<br>*Only common tests should be described solely by name; describe more complex techniques in the Methods section.*
☒	☐	A description of all covariates tested
☒	☐	A description of any assumptions or corrections, such as tests of normality and adjustment for multiple comparisons
☐	☒	A full description of the statistical parameters including central tendency (e.g. means) or other basic estimates (e.g. regression coefficient) AND variation (e.g. standard deviation) or associated estimates of uncertainty (e.g. confidence intervals)
☐	☒	For null hypothesis testing, the test statistic (e.g. *F*, *t*, *r*) with confidence intervals, effect sizes, degrees of freedom and *P* value noted<br>*Give P values as exact values whenever suitable.*
☒	☐	For Bayesian analysis, information on the choice of priors and Markov chain Monte Carlo settings
☒	☐	For hierarchical and complex designs, identification of the appropriate level for tests and full reporting of outcomes
☒	☐	Estimates of effect sizes (e.g. Cohen's *d*, Pearson's *r*), indicating how they were calculated

Our web collection on statistics for biologists contains articles on many of the points above.

Software and code

Policy information about availability of computer code

Data collection	Data were collected and recorded in Microsoft Excel, licensed for use to Oregon Health & Science University.
Data analysis	Data were analyzed by Graphpad Prismv7, licensed to users at Oregon Health & Science University.

For manuscripts utilizing custom algorithms or software that are central to the research but not yet described in published literature, software must be made available to editors and reviewers. We strongly encourage code deposition in a community repository (e.g. GitHub). See the Nature Portfolio guidelines for submitting code & software for further information.

Data

Policy information about availability of data

All manuscripts must include a data availability statement. This statement should provide the following information, where applicable:

- Accession codes, unique identifiers, or web links for publicly available datasets
- A description of any restrictions on data availability
- For clinical datasets or third party data, please ensure that the statement adheres to our policy

Raw data files are included with this submission. The manuscript has a data availability statement.

Research involving human participants, their data, or biological material

Policy information about studies with human participants or human data. See also policy information about sex, gender (identity/presentation), and sexual orientation and race, ethnicity and racism.

Reporting on sex and gender	n/a
Reporting on race, ethnicity, or other socially relevant groupings	n/a
Population characteristics	n/a
Recruitment	n/a
Ethics oversight	n/a

Note that full information on the approval of the study protocol must also be provided in the manuscript.

Field-specific reporting

Please select the one below that is the best fit for your research. If you are not sure, read the appropriate sections before making your selection.

[x] Life sciences [ ] Behavioural & social sciences [ ] Ecological, evolutionary & environmental sciences

For a reference copy of the document with all sections, see nature.com/documents/nr-reporting-summary-flat.pdf

Life sciences study design

All studies must disclose on these points even when the disclosure is negative.

Sample size	Previous publications had shown significant reductions in SARS-CoV-2 replication in lung lobes and lung inflammation in rhesus macaques following administration of a cocktail of human antibodies 24h after infection, using 4 animals per group (Van Rompay et al. PLOS Pathogens 17(7): e1009688 (2021). By testing a control anti-RSV antibody both pre-exposure and post-exposure, we had 8 animals in the combined control group. The purpose of the study was to determine whether aerosol delivery would be as effective as parenteral delivery, testing several different times of administration. This study design of n=4 per group allowed us to maximize the information gained.<br>We predicted that the infectious virus would be reduced from 5 log10 in controls to 0-1 log10 in treated animals (reductions of 3-4 log10) in the lung, thus showing significance with n=4 per group.
Data exclusions	No data were excluded from the analyses
Replication	Individual assays to quantify antibodies and virus were replicated using duplicate samples on different days.
Randomization	Animals were assigned as they were available within the age and weight range and sex was not considered.
Blinding	Persons performing ELISA analyses were blinded to the sample identification.

Reporting for specific materials, systems and methods

We require information from authors about some types of materials, experimental systems and methods used in many studies. Here, indicate whether each material, system or method listed is relevant to your study. If you are not sure if a list item applies to your research, read the appropriate section before selecting a response.

Materials & experimental systems

n/a	Involved in the study	
[ ]	[x]	Antibodies
[ ]	[x]	Eukaryotic cell lines
[x]	[ ]	Palaeontology and archaeology
[ ]	[x]	Animals and other organisms
[x]	[ ]	Clinical data
[x]	[ ]	Dual use research of concern
[x]	[ ]	Plants

Methods

n/a	Involved in the study	
[x]	[ ]	ChIP-seq
[x]	[ ]	Flow cytometry
[x]	[ ]	MRI-based neuroimaging

Antibodies

Antibodies used	mouse anti-SARS CoV2 N Genetex GTX635712

	mouse anti-SARS CoV2 S Genetex GTX632604<br>Horseradish peroxidase-conjugated goat anti-human IgG F(ab)2 fragment-specific polyclonal antibody (Jackson ImmunoResearch catalog no. 109-035-006).<br>SARS CoV-2 human mAbs CoVIC-96, AR-703, AR-720 (also called 1213H7)<br>RSV human mAb 25P13
Validation	CoVIC-96 and the RSV mAb 25P13 were produced under contract at Zalgen. AR-703 and AR-720 mAbs were produced under contract to Aridis Pharmaceuticals. Purity was assessed by SDS-PAGE and shown to be greater than 95% heavy and light chain. Endotoxin levels were less than 0.125 EU/mL. Specificity of CoVIC-96 was determined by binding to full length SARS CoV-2 Spike protein expressed in HEK-293T/17 cells and lack of binding to cells transfected with vector control. Prior to any in vivo work, activity and specificity of each mAb (AR-703, AR-720 and 25P13) were confirmed by antigen specific ELISA and SARS-CoV-2 focus-forming assays.

Eukaryotic cell lines

Policy information about cell lines and Sex and Gender in Research

Cell line source(s)	Vero E6-TMPRSS2-T2A-ACE2: obtained through BEI Resources, NIAID, NIH, cat. no. NR-54970<br>Zalgen used ExpiCHO cells for production of human mAbs for the study
Authentication	Vero cells obtained directly from BEI and not further authenticated; ExpiCHO cells were used at Zalgen and there is no information on authentication.
Mycoplasma contamination	Negative for mycoplasma
Commonly misidentified lines<br>(See ICLAC register)	None of these were used

Animals and other research organisms

Policy information about studies involving animals; ARRIVE guidelines recommended for reporting animal research, and Sex and Gender in Research

Laboratory animals	This study included 22 (12 male, 10 female) juvenile (2-3 years old) Indian-origin rhesus macaques (Macaca mulatta) ranging in weight from 3.5 – 5.65 kg at the study endpoint. The animals were housed at the Oregon National Primate Research Center (ONPRC) in an ABSL-2 facility for non-infectious studies and in an ABSL-3 biocontainment facility for all SARS-CoV-2 in vivo work. All animals were specific pathogen-free and tested negative for SARS-CoV-2 prior to inclusion in the study. Animals were socially housed or provided protected contact with conspecifics during the study.
Wild animals	No wild animals were used in this study
Reporting on sex	Sex as a variable was not considered for this study. SARS-CoV-2 infection in Macaca mulatta has been studied by many groups and is an acute infection characterized by virus replication and lung pathogenesis. No differences in sex have been observed in this virus infection model. As stated in the manuscript, the availability of animals was extremely tight, and the study team accepted healthy animals as they became available in groups of 4, without regard to sex.
Field-collected samples	There were no field-collected samples
Ethics oversight	All macaques in this study were managed according to the ONPRC animal care program, which is fully accredited by AAALAC International and is based on the laws, regulations, and guidelines set forth by the United States Department of Agriculture (e.g., the Animal Welfare Act and Regulations), Institute for Laboratory Animal Research (e.g., Guide for the Care and Use of Laboratory Animals, 8th edition), and the Public Health Service Policy on Humane Care and Use of Laboratory Animals. All animal and laboratory work was reviewed and approved by the Oregon Health and Science University (OHSU) Institutional Biosafety Committee (IBC) and the OHSU West Campus Institutional Animal Care and Use Committee (IACUC).

Note that full information on the approval of the study protocol must also be provided in the manuscript.
